# Microbiological Impact of Antimicrobial Photodynamic Therapy in Non-Surgical Periodontal Treatment

**DOI:** 10.3390/pharmaceutics17081070

**Published:** 2025-08-19

**Authors:** Filipa Passos Sousa, Mariana Anselmo Assunção, Lucinda J. Bessa, Ricardo Castro Alves

**Affiliations:** Egas Moniz Center for Interdisciplinary Research (CiiEM), Egas Moniz School of Health & Science, 2829-511 Almada, Portugal; fsousa@egasmoniz.edu.pt (F.P.S.); massuncao@egasmoniz.edu.pt (M.A.A.)

**Keywords:** periodontitis, photodynamic therapy, oral microbiome, non-surgical treatment

## Abstract

Periodontitis is one of the most common inflammatory diseases and it is linked to the presence of a dysbiotic subgingival microbiome. The purpose of this review is to evaluate the impact of antimicrobial photodynamic therapy (aPDT) on the subgingival microbiome. Herein, based on an extensive evaluation of randomized controlled trials (RCTs), the effects of aPDT as a supplement to non-surgical periodontal therapy (NSPT) were found to be the main focus of these works. Studies that focused on analyzing microbiological results were selected, yielding contradictory results. The observed microbiological changes were variable, even though some studies showed notable improvements in clinical indicators such as bleeding on probing (BOP), clinical attachment level (CAL), and probing depth (PD). Several studies found that aPDT did not significantly reduce important periodontal pathogens such *Tannerella forsythia*, *Porphyromonas gingivalis*, and *Aggregatibacter actinomycetemcomitans*. Nevertheless, after multiple aPDT sessions, other studies reported positive changes in the subgingival microbiome, with a rise in beneficial bacteria and a decrease in periodontopathogens. While aPDT seems to be a safe and well-tolerated adjuvant to non-surgical periodontal therapy, there is still conflicting evidence regarding how well it modulates the subgingival microbiota. Additional long-term research with larger sample sizes is required to evaluate the microbiological and clinical advantages of aPDT.

## 1. Introduction

Periodontal disease affects approximately 20–50% of individuals worldwide and is considered a significant public health concern. Its prevalence is projected to increase in the coming years, largely due to the aging global population [1].

Periodontitis is a chronic condition characterized by the accumulation of biofilms, which can lead to progressive destruction of the tissues surrounding the teeth, including the periodontal ligament and bone [2]. Complex and dynamic interactions between particular bacterial infections and harmful host immune responses are involved in the disease [2,3].

The oral microbiome is a diverse and dynamic community of microorganisms, including bacteria, fungi, viruses, protozoa, and archaea that reside in the oral cavity [1]. The complexity of the community can vary according to the niche within the oral cavity (teeth, gingival sulcus, tongue, lips, and palate). Oral microorganisms are organized in biofilms, surface-associated communities embedded in a self-produced extracellular polymeric substance (EPS) matrix that are highly structured physically and functionally [4,5].

Gram-negative bacteria from the red complex, *Porphyromonas gingivalis*, *Tannerella forsythia*, and *Treponema denticola*, are usually found in deep pockets in patients with periodontitis [2,6,7]. Although these bacteria are well-known periodontopathogens, new pathogens such as *Desulfobulbus* spp. (Gram-negative anaerobe), *Filifactor alocis* (Gram-positive anaerobe), and species of the phylum Candidatus Saccharibacteria have also recently been associated with periodontitis [8].

The development of periodontal disease has been linked to shifts in the relative abundance of microbial species, which disrupts the equilibrium of host–microbe interactions and triggers inflammatory changes [9]. Therefore, microbiome-targeted treatments to prevent periodontitis may be developed as a result of a better knowledge of the ecological processes and microbial interactions that facilitate progression to a dysbiotic state [6,10].

**Figure 1:**
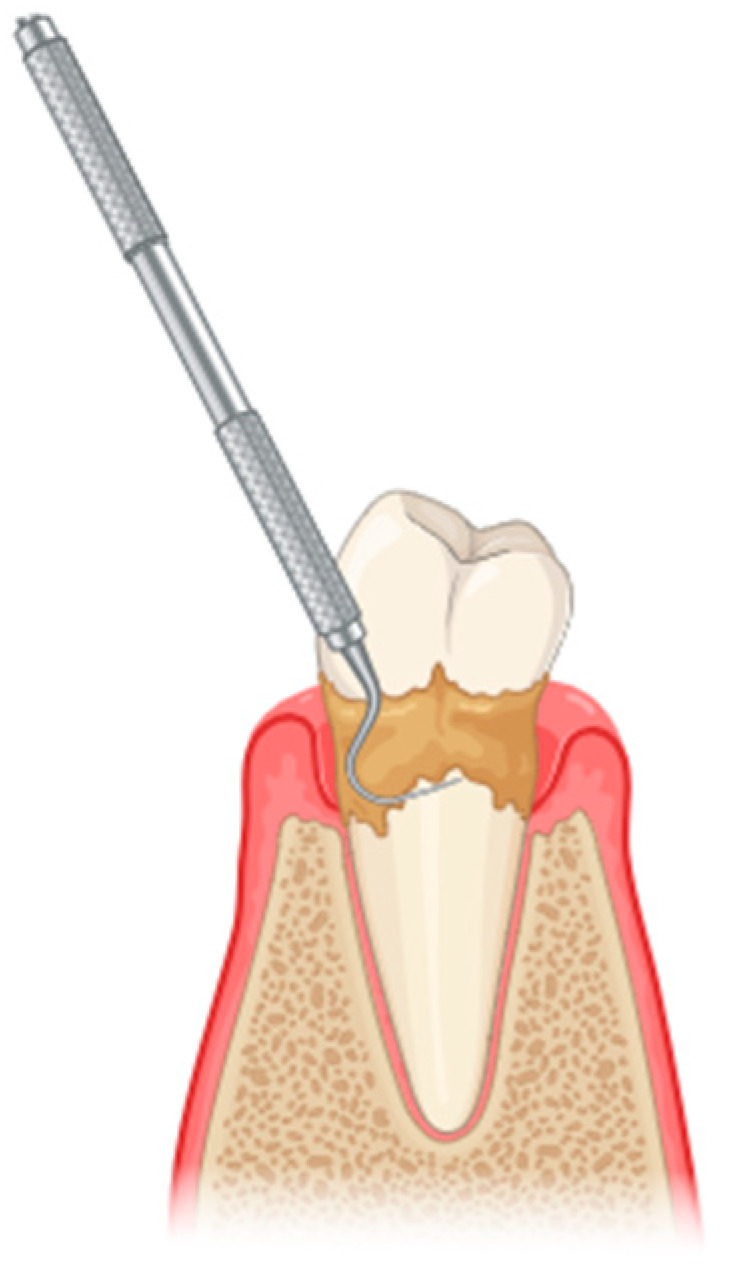
Scaling and root planing to remove plaque and calculus from the tooth surfaces (created with BioRender.

The initial treatment for periodontitis typically involves scaling and root planing (SRP), a procedure classified under non-surgical periodontal treatment (NSPT) (Figure 1). However, NSPT has several limitations, including anatomical challenges such as root concavities, deep pockets, furcation lesions, complex restorations contours, and limited tooth accessibility, among others [11]. Conventional mechanical periodontal therapy may be supplemented by several adjunctive methods [12]. The advent of laser technology in the healthcare domain, particularly in the context of the oral cavity, has allowed researchers and clinicians to conceptualize novel approaches to the treatment and management of oral diseases [13].

Photodynamic therapy has been proposed as an adjuvant in non-surgical periodontal treatment, based on findings from multiple studies [14]. The antibacterial effects of diode laser irradiation are primarily attributed to the thermal mechanisms and absorption of incident energy by the pigmented cell membranes of many periodontopathogenic bacteria [15].

Oscar Raab, a medical student in Munich (Germany), accidently discovered the principle of photodynamic treatment (PDT) in 1900 while working on his PhD thesis examining the toxicity of the dye acridine red on *Paramecium* spp. [16]. Raab’s supervisors, Von Tappeiner and Lesionek, used this procedure on tumor cells and published their results in 1905. For over 25 years, photodynamic cancer therapy has been used in dermatological clinics and hospitals around the world to treat disorders such as basal cell carcinoma and actinic keratosis [17]. Several medical specialties, including dermatology, oncology, gynecology, and urology, use PDT more frequently as a result of its beneficial therapeutic effects [18]. In addition to the treatment of chronic inflammation, photodynamic therapy offers an attractive alternative for the treatment of drug-resistant bacterial infections [19].

Numerous studies have investigated the treatment of periodontitis and its effects on the microbial composition within periodontal pockets. To analyze the microbiome, metagenomic approaches such as 16S ribosomal RNA (rRNA) gene sequencing and shotgun metagenomic sequencing have been widely adopted as practical and efficient techniques. These methods enable the identification of both cultivated and previously uncultivated bacterial species [3]. High-throughput DNA sequencing technologies have further facilitated the comprehensive profiling of oral microbial communities, providing detailed insights into the structure and diversity of microbial communities associated with periodontal health and disease [6].

**Figure 2:**
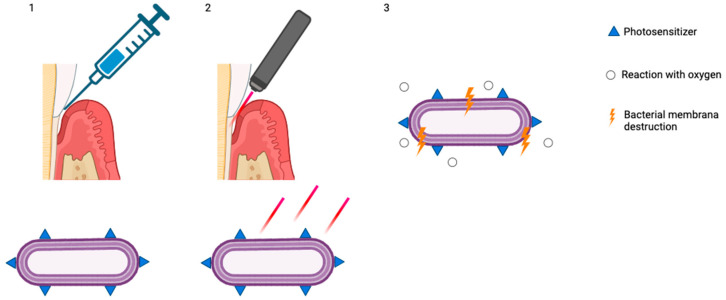
(**1**) Accumulation of photosensitizers on the bacterial membrane; (**2**) exposure and stimulation with red light; (**3**) reaction with oxygen and bacterial membrane destruction (created with BioRender.

The use of aPDT as an adjunct to non-surgical periodontal treatment (Figure 2) remains a subject of ongoing debate in the scientific community.

The studies in this review were gathered from a variety of databases, with the majority coming from PubMed and Scopus. A literature search was conducted using several keywords, including periodontitis, antimicrobial photodynamic therapy, and the oral microbiome. Randomized clinical trials published since 2009 were included.

## 2. In Vitro Efficacy of aPDT

In vitro tests have been carried out to find ways of enhancing the aPDT technique by adding other chemical compounds to the photosensitizer (Table 1). For instance, its combination with potassium iodide has shown efficacy in reducing microbial viability [20]. An in vitro study conducted by Bueno-Silva and collaborators evaluated the antimicrobial efficacy of aPDT plus methylene blue (MB) in reducing the metabolic activity and altering the microbial composition of multi-species oral biofilms. This study involved four groups: a control group (biofilm without treatment) and three test groups (LED: red light application without photosensitizer; MB: methylene blue application without light; and MB + LED: methylene blue application followed by red light irradiation). aPDT using MB and LED reduced the metabolic activity and total bacterial counts in multispecies subgingival biofilms. These results suggest that this approach may be an effective way of controlling polymicrobial oral biofilms [21].

The ability of aPDT to eliminate specific bacteria in vitro, particularly *Porphyromonas gingivalis*, has been demonstrated, with the therapy achieving approximately 96% bacterial reduction in the study by Fekrazad et al. [30]. Oruba and his colleagues also conducted an in vitro study to evaluate the effectiveness of aPDT against *P. gingivalis* in gingival fibroblasts and keratinocytes [22]. Their results also revealed a high capacity for bacterial elimination, suggesting its therapeutic potential for treating periodontal infections [22]. Similar results have been found in other in vitro studies involving this microorganism [23,24,25,26].

The in vitro effects of aPDT on other species, such as *Aggregatibacter actinomycetemcomitans*, have also been studied [27,28,29]. De Sousa et al. observed a reduction in *A. actinomycetemcomitans* activity of more than 90%, indicating that aPDT is an effective strategy for controlling this important periodontopathogenic bacterium [29].

## 3. Antimicrobial Effects of aPDT During Active Periodontal Therapy

Several clinical studies have shown limited or no microbiological benefits when aPDT was used as an adjunct to SRP (Table 2 and Table 3). As an example, Pulikkotil et al. observed significant clinical improvements following SRP and aPDT, but found no significant reduction in *A. actinomycetemcomitans* compared to SRP alone [31]. Similar findings were reported in randomized controlled trials (RCTs) involving both smokers and non-smokers, where microbial biomarkers such as *P. gingivalis*, *Prevotella intermedia*, and *Prevotella nigrescens* remained unaffected by aPDT, despite minor clinical benefits such as reduced bleeding on probing (BoP) [32,33,34].

In contrast, some studies have demonstrated positive microbiological outcomes with aPDT [35,36,37,38,39]. A trial by Pinheiro et al. reported a 14.66% greater bacterial reduction in periodontal pockets when aPDT was combined with SRP, suggesting an increased decontamination efficacy [35]. Furthermore, an RCT comparing single vs. multiple aPDT sessions found that repeated aPDT applications induced significant shifts in subgingival microbial composition, including a decrease in anaerobic bacteria and an increase in aerobic, health-associated species. These results highlight that the antimicrobial effectiveness of aPDT may vary depending on the specific protocol used [36].

Different bacterial susceptibilities to treatment procedures were also observed. One split-mouth trial found that aPDT was more effective than SRP in reducing *A. actinomycetemcomitans*, whereas SRP was more effective against red complex pathogens such as *P. gingivalis* and *T. forsythia*. This complementarity suggests a potential synergistic effect when both methods are combined, though recolonization, particularly by *T. forsythia*, was observed in aPDT-treated sites [37]. Another study found that four sessions of adjunctive aPDT led to a substantial decrease in pathogens from the red and orange complexes, reinforcing the notion that multiple applications may be more effective [38].

Therefore, the existing literature on the microbiological effects of aPDT in periodontitis presents heterogeneous and, at times, conflicting findings. This inconsistency likely reflects variation in study design, baseline disease severity, aPDT protocols (e.g., photosensitizers, light sources, treatment frequency), and microbiological analysis techniques. As such, the current evidence base warrants cautious interpretation, and generalized conclusions should be avoided. Moreover, rather than seeking to resolve this heterogeneity across studies, this review highlights it as a critical issue that underscores the need for standardized protocols and harmonized microbiological endpoints in future trials.

## 4. aPDT in Periodontal Maintenance

In the context of supportive periodontal therapy (SPT), where microbial control is critical to prevent recurrence, the role of aPDT appears more promising (Table 4 and Table 5). Several studies reported significant short-term reductions in specific pathogens [40,41,42]. For example, a study involving patients in maintenance therapy showed decreased levels of *Fusobacterium nucleatum* and *Eubacterium nodatum* at three months post-aPDT, although recolonization by other species such as *Eikenella corrodens* occurred at six months, highlighting the transient nature of microbiological improvements [42]. Similarly, Corrêa et al. observed a significant short-term reduction in *A. actinomycetemcomitans* within 3 and 7 days post-treatment in sites treated with SRP + aPDT [41]. However, no significant differences were observed in *P. gingivalis* levels between groups, suggesting species-specific responses to aPDT [41]. Another trial with repeated aPDT applications in SPT patients demonstrated greater reductions in *P. gingivalis*, *A. actinomycetemcomitans*, BoP, and the periodontal inflamed surface area (PISA) index compared to SRP alone, suggesting cumulative microbiological and clinical benefits with repeated therapy [43].

Grzech-Leśniak et al. demonstrated that multiple aPDT applications led to a significant decrease in several key periodontal pathogens, including *T. forsythia* and *T. denticola*, whereas SRP alone reduced only a subset of these species [44]. Interestingly, *A. actinomycetemcomitans* levels remained unchanged despite repeated aPDT applications, suggesting resilience or rapid recolonization by this pathogen [44]. In another RCT, Kolbe et al. evaluated aPDT as a monotherapy and found it to be as effective as SRP in reducing *A. actinomycetemcomitans* and *P. gingivalis* levels, proposing aPDT as a less invasive option for selected residual pockets [40].

On the other hand, not all studies support the adjunctive benefit of aPDT in the maintenance phase. Therefore, long-term effects remain a concern. A one year-long study involving multiple aPDT applications showed no significant differences in microbial profiles between the test and control groups across time points, indicating that aPDT, at least with the laser and photosensitizer protocols used, may not provide sustained microbiological benefits in residual pockets [45]. Similarly, a more recent RCT with 3-month follow-up found reductions in several key pathogens in both the SRP and SRP + aPDT groups, with no intergroup differences, once again casting doubt on the short-term extra microbiological advantages of aPDT [46].

The microbiological changes observed following aPDT extend beyond transient antimicrobial effects; they indicate a broader ecological restructuring of the subgingival biofilm. The rapid reduction in key periodontal pathogens, coupled with the disruption of biofilm architecture, facilitates recolonization by commensal species and promotes a rebalanced microbial ecosystem. However, the sustainability of these changes beyond six months remains uncertain, and aPDT should not be regarded as a shortcut to long-term remission. Unlike antibiotics, aPDT offers these advantages without contributing to antimicrobial resistance. Future research should incorporate advanced microbial profiling techniques and longer follow-up periods to determine whether these ecological shifts translate into sustained periodontal health.

## 5. Studies’ Limitations

In periodontal research specifically, the literature guidelines emphasize the importance of sample size calculation based on expected effect sizes, variances, and clinical endpoints; failing to achieve minimum thresholds compromises the validity of results and hinders reproducibility. Thus, while some studies observed reductions in key pathogens following aPDT [31,34,36,37,38,39,40,41,42,44,45,46], these findings must be interpreted with caution, particularly since insufficient sample sizes reduce precision, amplify random error, and undermine reliability of subgroup comparisons and microbiological trends.

Robust evaluation of the long-term efficacy and durability of aPDT requires large-scale randomized trials with extended follow-up, given that periodontitis is a chronic condition. However, several studies included in this review are limited by short follow-up durations (ranging from 1 to 6 months) [31,32,34,35,37,38,41,42,43,44,46] and small sample sizes (often fewer than 30 participants) [32,33,34,35,36,37,38], which present important methodological limitations. Only two studies [44,45] included more than 30 participants. Short-term follow-up may capture only transient improvements rather than sustained periodontal health, whereas the progression or recurrence of periodontitis typically unfolds over several months or years. Additionally, the inclusion of small cohorts raises concerns about statistical power and the generalizability of the findings. Limited sample sizes hinder the detection of modest yet clinically meaningful changes in microbial composition and reduce the reliability of subgroup analyses.

One of the most critical limitations is the wide variability in aPDT protocols across studies. Differences in photosensitizer types (curcumin, methylene blue, toluidine blue, and ICG), laser wavelengths (465–980 nm), energy dosimetry, number of applications (single vs. repeated sessions), and timing significantly impact the outcomes. Some photosensitizers have antibacterial properties, and this can lead to biased results in terms of the effectiveness of the therapy [47]. This lack of standardization makes cross-study comparisons difficult and may explain inconsistent microbiological results.

An important limitation of some aPDT studies is that certain photosensitizers display inherent antimicrobial activity, even in the absence of light. This ‘dark toxicity’ means that any reduction in microbial populations observed may not be due solely to photodynamic action, but could also be caused by the intrinsic antimicrobial effects of the photosensitiser itself. For instance, phenothiazine dyes such as methylene blue have cationic properties that allow them to disrupt microbial membranes even without light activation [40,48,49]. When studies fail to include adequate dark and light controls, such as untreated, photosensitiser-only, and light-only groups, it becomes difficult to distinguish between true photodynamic efficacy and non-light-dependent antimicrobial effects. Consequently, such experimental designs carry a risk of overestimating the contribution of light-mediated processes. Therefore, future aPDT research should rigorously implement dark/light control groups, and studies lacking these comparisons should be clearly identified to mitigate bias and ensure accurate attribution of antimicrobial outcomes.

Variability in microbial sampling techniques (e.g., paper points vs. curettes), detection methods (e.g., culture, qPCR, checkerboard DNA–DNA hybridization, metagenomics), and target species can contribute to the inconsistent findings reported in the literature. Most studies focus on a limited number of traditional periodontal pathogens (e.g., *P. gingivalis*, *T. forsythia*, *A. actinomycetemcomitans*), often overlooking the broader composition and structure of the oral microbiome. A narrow focus on a limited set of pathogens may underestimate the broader ecological impact of aPDT on the subgingival microbial community. While quantitative real-time PCR (qPCR) offers high sensitivity and specificity for predefined targets, such as *Porphyromonas gingivalis* and *Aggregatibacter actinomycetemcomitans*, its scope is inherently limited. In contrast, 16S rRNA gene sequencing provides a more comprehensive profile of complex biofilms, enabling the detection of low-abundance taxa and supporting in-depth ecological analyses [50,51]. These capabilities are essential for understanding community-level shifts, microbial diversity, and recolonization dynamics following aPDT. High-throughput sequencing should therefore be considered the reference standard in future aPDT studies, particularly those aiming to assess ecological outcomes. Additionally, the use of standardized reference databases and taxonomic frameworks enhances the comparability of findings across studies.

Some trials do not report double-blinding or the use of placebo treatments (e.g., photosensitizer or light without activation), potentially introducing bias in clinical and microbiological assessments.

## 6. Clinical Safety of aPDT in Periodontal Therapy

aPDT is considered a safe and well-tolerated addition to non-surgical periodontal treatment. According to the reviewed studies, there were no significant reports of tissue damage, allergic reactions to photosensitizers or systemic complications. Patients generally tolerate the procedure well, even when multiple sessions are required. When applied correctly, aPDT appears to be a safe adjunctive tool with minimal risks and good patient tolerance.

## 7. Conclusions

The use of aPDT as an adjunct to non-surgical periodontal treatment remains a subject of ongoing debate in the scientific community. While some evidence supports the microbiological benefits of aPDT, particularly when applied repeatedly or in specific contexts such as SPT, the overall data remains heterogeneous. Differences in treatment protocols, photosensitizers, light sources, and patient populations (e.g., smokers versus non-smokers, active treatment versus maintenance) can have a significant impact on outcomes.

Notably, aPDT appears to have species-specific effects and may reduce certain pathogens such as *A. actinomycetemcomitans* and *F. nucleatum* in the short term, which was not observed for *P. gingivalis.* The long-term stability of the microbial environment and the prevention of recolonization remain uncertain.

In order to better define the role of aPDT in affecting periodontal microbiota, future research should focus on identifying suitable target populations, optimizing application techniques, and demonstrating long-term effects.

Randomized controlled trials with follow-up periods of at least 12 months, adequately powered sample sizes, and protocol standardization (photosensitizer, wavelength, dosage) are needed to accurately evaluate the sustained microbial and clinical effects of aPDT. To improve the comparability and reliability of future aPDT research, it is recommended that standardized protocols for microbiome assessment be adopted. This includes the use of consistent sampling methods and the application of high-resolution sensitive molecular techniques such as 16S rRNA gene sequencing to enhance detection and quantification accuracy. Furthermore, transparent reporting of methodological protocols and validation procedures should be strongly encouraged. Establishing these best practices will support more robust and reproducible evaluations of aPDT efficacy on microbial communities.

## Figures and Tables

**Table 1:** Efficacy of aPDT—in vitro oral biofilm studies.

Photosensitizer/Treatment	Bacterial Species	Outcomes	Study Reference
Methylene Blue + Potassium Iodide	Oral biofilm	KI potentiates MB-aPDT efficacy against oral biofilms	[20]
Methylene Blue	Multi-species oral biofilm	Significant reduction in biofilm viability	[21]
Not specified	*P. gingivalis*	aPDT effectively reduces *P. gingivalis* in host cells	[22]
Radachlorin and Toluidine Blue O	*P. gingivalis*	Both photosensitizers effective in *P. gingivalis* inactivation	[23]
Sinoporphyrin Sodium + LED (390–400 nm)	*P. gingivalis*	Significant bacterial reduction	[24]
Phycocyanin only; Diode Laser only; Phycocyanin + Diode Laser (aPDT)	*P. gingivalis*	aPDT group showed the greatest microbial reduction	[25]
Dendrosomal Curcumin + Blue Laser	*P. gingivalis*	Strong antibacterial effect observed	[26]
Various photosensitizers and protocols	*P. gingivalis*, *A. actinomycetemcomitans*, others	aPDT is effective in reducing key periodontopathogens	[27]
Curcumin-based aPDT vs. Clorohexidine	*A. actinomycetemcomitans*	Comparable effects	[28]
Not specified	*A. actinomycetemcomitans*	Proper physical and chemical settings enhance aPDT efficacy	[29]
Indocyanine Green (Emundo) + Laser (aPDT)	*P. gingivalis*	Significant antibacterial effect comparable to chemical agents	[30]

**Table 2:** Effects of adjunctive aPDT in NSPT.

Bacterial Taxa	Outcome Observed	Study Reference
*Aggregatibacter actinomycetemcomitans*	Control Group, 4.30 ± 0.86 Teste Group, 4.88 ± 0.96 After 3 months, the reduction in periodontopathogens of the test group was greater but with no statistically significant difference	[31]
*Aggregatibacter actinomycetemcomitans* *Porphyromonas gingivalis* *Prevotella intermedia* *Tannerella forsythia* *Treponema denticola* *Peptostreptococcus micros* *Fusobacterium nucleatum* *Campylobacter rectus* *Eubacterium nodatum* *Eikenella corrodens* *Capnocytophaga* spp.	No statistically significant difference was observed between the two treatment groups	[32]
*40 bacterial species*	SRP or SRP + aPDT was not able to reduce bacterial levels in smokers	[33]
*Aggregatibacter actinomycetemcomitans* *Porphyromonas gingivalis* *Prevotella intermedia* *Tannerella forsythia* *Treponema denticola*	The reduction in periodontopathogens of the test group was greater, but with no statistically significant difference	[34]
The study did not provide a detailed list of the specific bacterial species evaluated	SRP, 81.24% reduction SRP + aPDT, 95.90% reduction with statistically significant difference	[35]
*Firmicutes*, *Actinobacteria*, *Proteobacteria*, *Bacteroidetes*, *Fusobacteria*, *Spirochaetae*, and *Synergistetes*, *Streptococcus*, *Actinomyces*, *Porphyromonas*, *Fusobacterium*, *Rothia*, *Lautropia*, *Neisseria*, *Treponema_2*, *Capnocytophaga*, *Leptotrichia*, *Haemophilus*, *Fretibacterium*, *Prevotella* and *Veillonella*	The proportion of aerobic bacteria increased more while anaerobic bacteria decreased more in the repeated-PDT group with statistically significant difference	[36]
Forty bacterial species	SRP was more effective against *P. gingivalis* and *T. forsythia*; aPDT was more effective against *A. actinomycetemcomitans*	[37]
Red and Orange Complex Pathogens	Microbial profiles were affected in SRP + aPDT group (four sessions) with statistically significant difference	[38]
*P. gingivalis*	Significant improvements in clinical and microbiological parameters	[39]

**Table 3:** Methodological parameters in studies on aPTD during active periodontal therapy.

Design	Sample Size	PS	Wavelenght	Sessions	DNA Test	Follow-Up	Study Reference
Split-mouth RCT	20	MB	628 Hz	1	rPCR	3 m	[31]
Parallel-arm RCT	24	TB	670 nm	1	NR	6 m	[32]
Split-mouth RCT	20	Curcumin	NR	1	DNA–DNA hybridization technique	12 w	[33]
Parallel RCT	24	TB	625–635 nm	2	PCR	3 m	[34]
Non-randomized clinical	10	TB	632.8 nm	1	Homogenization, dilution, seeding and cultivation	Immediate	[35]
Split-mouth RCT	17	MB	650–670 nm	Repeated	16S rRNA gene sequencing	NR	[36]
Split-mouth RCT	10	HELBO Blue Photosensitizer^®^	660 nm	1	DNA-DNA hybridization technique	3 m	[37]
Split-mouth RCT	20	NR	670 nm	4	DNA-DNA hybridization technique	3 m	[38]
Split-mouth RCT	54	MB	660 nm	1	Oligonucleotide probe		[39]

**Table 4:** Effects of aPDT on periodontal pathogens during SPT.

Outcome Observed	Bacterial Taxa	Study Reference
Significant reduction at 3 months	*A. actinomycetemcomitans*	[40]
Significant reduction at 3 and 6 months (earlier effect)	*P. gingivalis*	
Significant reduction within 7 days post-treatment with SRP + aPDT; no change with SRP alone	*A. actinomycetemcomitans*	[41]
No significant difference between SRP + aPDT and SRP alone	*P. gingivalis*	
Significant reduction at 3 months;	*F. nucleatum*	[42]
Decreased at 3 months; recolonization at 6 months	*Eubacterium nodatum*	
Greater reductions in *P. gingivalis*, *A. actinomycetemcomitans*, with repeated aPDT applications compared to SRP alone	General Microbial Profile	[43]
Significant decrease with multiple aPDT applications; no change with SRP alone	*T. forsythia* *T. denticola*	[44]
No change with multiple aPDT applications; rapid recolonization observed	*A. actinomycetemcomitans*	
No significant difference	*A. actinomycetemcomitans* *T. denticola* *T. forsythia* *P. gingivalis*	[45]
Significant reduction by 3 months, but without significant intergroup (test and control) difeferences	*A. actinomycetemcomitans* *P. gingivalis* *T. forsythia* *F. nucleatum*	[46]

**Table 5:** Methodological parameters in aPDT periodontal maintenance studies.

Design	Sample Size	PS	Wavelenght	Sessions	DNA Test	Follow-Up	Study Reference
Split-mouth RCT	22	MB	660 nm	1	PCR	6 m	[40]
Split-mouth RCT	15	MB	660 nm	1	PCR	3 m	[41]
Split mouth RCT	24	HELBO Blue Photosensitizer^®^	670 nm	1	PCR	6 m	[42]
Split mouth RCT	24	IDG	909 nm	2	PCR	6 m	[43]
Parallel RCT	40	TB	635 nm	3	PCR	6 m	[44]
Parallel RCT	34	MB	660 nm	NR	PCR	12 m	[45]
Split mouth RCT	24	IDG	810 nm	Multiple	PCR	3 m	[46]

## Data Availability

This article does not report any original data, and the research described is based on a review of existing literature.

## Abbreviations

The following abbreviations are used in this manuscript:

aPDT	Antimicrobial Photodynamic Therapy
NSPT	Non-surgical Periodontal Therapy
RCT	Randomized Controlled Trial
BOP	Bleeding on Probing
CAL	Clinical Attachment Level
PD	Probing Depth
SRP	Scaling and Root Planing
RNA	Ribonucleic Acid
DNA	Deoxyribonucleic Acid
MB	Methylene-Blue
LED	Light-emitting Diode
SPT	Supportive Periodontal Therapy
PISA	Periodontal Inflamed Surface Area
PCR	Polymerase Chain Reaction
